# Prevention of Postoperative Urethral Strictures by Irrigation with 5-Fluorouracil via a Modified Urinary Catheter

**DOI:** 10.3390/medicina60010102

**Published:** 2024-01-05

**Authors:** Yerbol Kairambayev, Tolkyn Bulegenov, Nazarbek Omarov, Muratkan Kuderbayev, Marat Syzdykbayev, Natalya Glushkova, Dinara Akhmetzhanova, Alida Kaskabayeva, Zhanna Muzdubayeva, Kuat Akimzhanov, Lyudmila Pivina

**Affiliations:** 1 Department of Surgery Disciplines, Semey Medical University, Semey 071400, Kazakhstan; kayrambaev-11@mail.ru (Y.K.); tolkynbul@mail.ru (T.B.); nazarbek.omarov@smu.edu.kz (N.O.); kuderbaevm@mail.ru (M.K.); kuat73@inbox.ru (K.A.); 2 Department of Anesthesiology and Reanimatology, Semey Medical University, Semey 071400, Kazakhstan; fortunato74@mail.ru; 3 Health Policy and Organization Department, Al-Farabi Kazakh National University, Almaty 050040, Kazakhstan; natalya.glushkova@kaznu.edu.kz; 4 Department of Pediatrics, Semey Medical University, Semey 071400, Kazakhstan; dinara.akhmetzhanova@smu.edu.kz; 5 Department of Internal Medicine, Semey Medical University, Semey 071400, Kazakhstan; alida.71@mail.ru (A.K.); muzduba@mail.ru (Z.M.); 6 Department of Emergency Medicine, Semey Medical University, Semey 071400, Kazakhstan

**Keywords:** ureteral stricture, 5-fluorouracil, modified catheter, prevention, benign prostatic hyperplasia, surgery

## Abstract

*Background and Objectives:* Urethral strictures are the most common complications after surgical treatments of benign prostatic hyperplasia (BPH). Despite various preventive measures, the search for medications with antiproliferative activity and the development of surgical procedures to prevent the development of urethral strictures are still relevant. We evaluated the preventive efficacy of 5-fluorouracil against urethral strictures in patients undergoing surgery for BPH. *Materials and Methods:* A non-randomized clinical trial including 246 male patients with an average age of 70.0 ± 8.0 years was conducted. The main study group included 124 patients who, in addition to the standard treatment, received lavage with a 5-fluorouracil solution (1000 mg/20 mL per 500 mL of 0.9% isotonic saline) using a modified three-way urethral catheter. The monitoring of clinical, laboratory, and instrumental parameters was carried out 10 days, 3 months, and 6 months after surgery. *Results:* The evaluation of severity for dysuria symptoms in patients using the IPSS scale throughout the entire follow-up period showed a statistically significant decrease in ischuria and stranguria, prolongation of the interval between urinations, a decrease in intermittent urination, urinary incontinence, and straining before urination in the main group in comparison with the control patients. The patients of both study groups noted an improvement in the quality of life. It was found statistically significant decrease in the maximum urinary flow rate in the main group (*p* < 0.001). In the control group, after three months, four cases of urethral strictures and stenosis were recorded; after six months, this rate reached nine cases (7.3%), while in the main group, only one patient with infravesical obstruction was found (0.8%) (χ^2^ = 3.855, *p* < 0.05). *Conclusions:* The results of our study could indicate the effectiveness of the antiproliferative drug 5-fluorouracil in combination with use of a modified catheter in relation to the development of postoperative urethral strictures.

## 1. Introduction

Benign prostatic hyperplasia (BPH) is one of the most typical diseases among middle-aged and elderly men; it is characterized by long-standing symptoms from the lower parts of the urinary tract, which lead to violations of physiological functions and a decrease in functional well-being [1]. About 26% of men aged 40 to 49 years have severe symptoms in the lower part of the urinary tract (>7 points according to the IPSS scale); at the age of more than 70 years, this indicator increases by up to 46%. Most of them have symptoms of dysuria [2,3]. Currently, several methods of treatment are available for patients with BPH, including drug treatment, surgical intervention in the form of open prostatectomy, transurethral resection of the prostate (TURP), and minimally invasive treatment methods, such as laser and microwave therapy [4,5].

Urethral stricture is one of the frequent pathologies in urology, which can lead to the obstructive dysfunction of urination and long-term damage to the entire urinary tract. According to the definition of the leading experts of the International Urological Council of the International Society of Urologists (SIU/ICUD, 2014), urethral stricture is a pathological narrowing of any part of the urethra surrounded by a corpus spongiosum due to the development of spongiofibrosis [2]. Urethral stricture is a common and complex urological pathology. The incidence has been steadily increasing in recent decades; it varies from 0.6% to 0.9% in the population and depends on the age of the patients. Extended and subtotal lesions have been diagnosed in 15–18% of all cases [6,7]. The average individual cost per patient with stricture disease is three times higher than for other urological pathologies [1]. In countries with a high level of medical development, strictures of iatrogenic etiology are most common due to the widespread use of transurethral endoscopic operations [3].

The pathogenesis of urethral stricture is based on damage to the epithelium of the urethra, followed by squamous metaplasia, changes in the extracellular matrix of the corpus spongiosum of the urethra, and the development of spongiofibrosis [8]. The normal connective tissue of the wall of the urethra is replaced by dense fibrous tissue with a decrease in the ratio of collagen types I and III. These changes lead to a decrease in the proportion of smooth muscle tissue and collagen in the corpus spongiosum and, as a result, the inhibition of nitric oxide synthesis in the area of the stricture, tissue hypoxia, and scar progression [9].

For the treatment of strictures, surgical methods are usually used and, in some cases, agents with an antiproliferative effect, such as corticosteroids (triamcinolone, methylprednisolone) [10]. However, an obstacle to such treatments is resistance to corticosteroids, which has become increasingly common in recent years [11]. This situation leads to the need to use other medications with an antiproliferative effect. Antitumor medication 5-fluorouracil has such properties.

The aim of our study is to evaluate the preventive efficacy of 5-fluorouracil against urethral strictures and associated complications in patients undergoing surgery for BPH.

## 2. Methods

### 2.1. Characteristics of the Object of Study

A non-randomized clinical trial including 246 male patients aged 50–80 years who underwent adenomectomy or TURP was conducted. Exclusion criteria were patients with urethral stricture diagnosed before surgery, as well as patients with established malignant prostatic hyperplasia, acute renal injury or chronic renal failure, and heart failure of functional classes III–IV. Patients with previous strokes, parkinsonism, severe cerebral atherosclerosis, and other diseases accompanied by severe neuromuscular dysfunction of the bladder were also not included in the study. Only persons with compensated diseases underwent surgery.

The distribution of the study participants into the study group and the control group was carried out through the main investigator taking into account the compatibility of socio-demographic indicators and inclusion and exclusion criteria in the order of admission of patients for surgical treatment. Thus, each patient had an equal chance of being included in both study groups.

The sample calculations were carried out using the Sample Size Calculator [12]. Of the operated patients, 131 patients underwent TURP, and 115 underwent open adenomectomy.

The average age of the study participants was 70.0 ± 8.0 years. About a quarter of patients lived in rural areas and received a referral for surgical treatment from a doctor in district hospitals. Only 18.7% of patients were referred for treatment by outpatient urologists; approximately the same number of patients initially applied to the ambulance service. Type 2 diabetes mellitus, arterial hypertension, and heart failure (I-II functional classes by NYHA) were registered in 15.4%, 37.8%, and 8.53% of patients, respectively (Table 1).

The main study group included 124 patients who, in addition to the standard postoperative treatment (antibiotic therapy, hemostatic therapy, antispasmodics, analgesics, alpha-blockers, 5-alpha reductase inhibitors), received prophylactic intervention after surgery in the form of lavage with a 5-fluorouracil solution using a modified three-way catheter. The duration of such treatment was up to 10 days. The control group consisted of 122 patients matched by age, social status, place of residence, and body mass index (BMI), who also underwent surgery for BPH. In the control group, after surgery, standard treatment was carried out. Bladder lavage was performed with furacillin using a Foley catheter.

Monitoring of clinical, laboratory, and instrumental parameters was carried out 10 days, 3 months, and 6 months after the surgery. Patients were warned about the need for repeated examination over time to prevent possible complications. We studied the severity of urination disorders symptoms and estimated the patients’ quality of life in accordance with the IPSS scale.

### 2.2. Ethical Approval Details

The study was approved by the Local Ethics Commission of the Semey Medical University on 28 December 2018; Protocol N 4. All participants signed an informed consent form.

### 2.3. Protocol for Transurethral Resection of BPH

After treating the surgical field under anesthesia, urethrocystoscopyis performed to determine the presence of prostate adenoma and its size, tuberosity, and density. Transurethral resection of the middle and lateral lobes of the prostate was performed. The volume and weight of the resected tissue were determined, which were then sent for histological examination. The bladder was catheterized through the urethra with a Foley catheter, and the balloon on the Foley catheter was inflated for hemostatic purposes. The surgical operation lasted for about one hour on average. Five urologists from two hospitals of Semey City perform such types of surgical interventions. All of them have more than 10 years of experience as surgeons and are highly qualified. A prerequisite for their work is internships in central republican clinics and foreign clinics. Resectoscopes of 24 Charriere calibers were used.

### 2.4. Description of the Utility Model of the Modified Three-Way Foley Catheter

A utility model of a modified three-way Foley catheter (utility model patent No. 4223 dated 9 August 2019) [13] was used for bladder drainage followed by urethral lavage with antiseptic solutions in the early and late postoperative periods. It is an improved model of the three-way Foley catheter [14].

**Figure 1:**
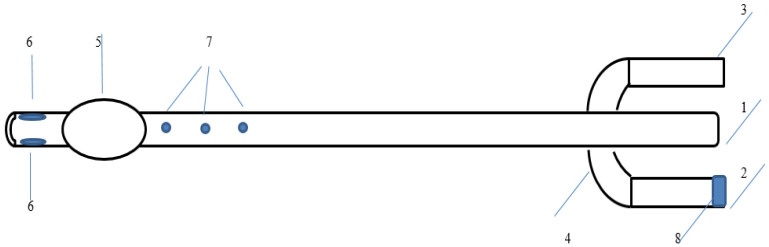
A utility model of a modified three-way Foley catheter. 1: Drainage funnel, 2: Channel for balloon inflation, 3: Channel for the introduction of drugs or antiseptics, 4: Anti-return valve, 5: Balloon for fixing the catheter in the bladder, 6: Drainage channels, 7: Irrigation channels for washing the urethra, 8: Valve cover.

The catheter has three channels: (1) one for administering medications and irrigating the urethra, (2) one for removing urine and washing the bladder cavity, and (3) another for inflating the balloon. There are three holes at the distal end of the catheter: two of them are used for removing urine and rinsing the bladder, and one is for introducing drugs into the urethra and irrigating it. Inside the catheter, the passages are separated, which eliminates the possibility of mixing drugs with urine during the administration of drugs during urethral irrigation. The catheter flushes the bladder through the first main channel and the urethra through a modified third channel. At the distal end, the pre-existing opening of the irrigation channel is closed with medical silicone glue so that the drug for urethral irrigation does not enter the bladder (Figure 1).

### 2.5. Description of Preventive Intervention

Patients of the study group were administered 1000 mg/20 mL of 5-fluorouracil per 500 mL of 0.9% sodium chloride solution into the urethra and bladder using a modified three-way urethral catheter. Irrigation of the urethra and bladder with a solution of 5-fluorouracil was carried out from the first day after surgery for 5 to 10 days and took about 3 min.

### 2.6. Instrumental Methods of Examination

Transabdominal ultrasound examination of the prostate with the determination of the volume of residual urine and volume of prostate gland was performed before surgery, as well as 10 days, 3 months, and 6 months after surgery.

To conduct uroflowmetry, we used the UFM-01 YAROVIT uroflowmeter, which is a special funnel that characterizes urination. The device is connected to a personal computer. Sensors record the duration and volumetric flow rate of urine. A graph is constructed based on the dynamics of the characteristics, and the results are displayed on the monitor. The study was conducted before surgery, as well as 10 days, 3 months, and 6 months after surgical treatment. A decrease in the maximum urine flow rate indicates the presence of a bladder outlet obstruction of the urethra.

In the case of a decrease in urodynamic parameters and an increase in the volume of residual urine, posterior irrigation ureteroscopy and cystourethrography were performed to determine the presence of urethral strictures or stenosis.

### 2.7. Evaluation of the Severity of Dysuric Symptoms and Quality of Life in Dynamics

To assess the dynamics of the severity of dysuric symptoms, we used the IPSS scale (International Total Scoring System for Prostate Diseases). As an additional method, the definition of the quality of life index, determined in points, was used. Number of points: from 0 to 7 indicates minor disorders, from 8 to 19—moderate disorders, and from 20 to 35 indicates severe symptoms of the disease.

### 2.8. Statistical Data Processing

For categorical variables, data were given as absolute and relative numbers. For qualitative data, the significance of differences in groups was determined by performing the Chi-square test (χ^2^). For quantitative data, central trends were measured. All quantitative variables were distributed with a deviation from the normal distribution. For quantitative data, given that the distribution was non-normal, the result was presented as the median and 25–75 percentiles. Calculations of the significance of differences were made using the Mann–Whitney test. The assessment of within-group dynamics was performed using the Friedman test for K-related samples with repeated measures. The critical level of significance of differences in the groups was taken as *p* < 0.05. All statistical analysis procedures were performed using the SPSS 20 program (IBM, Armonk, NY, USA).

## 3. Results

Most patients before surgery received conservative treatment for a year or more; in almost a third of patients, the operation was performed within a year from the onset of clinical symptoms. 42.3% of patients with acute symptoms of urinary retention were admitted on an emergency basis. With regard to preoperative status, social characteristics, and the presence of type 2 diabetes mellitus in the main and control groups, no statistically significant differences were found.

Comparison of laboratory parameters in the study groups indicated the presence of statistically significant differences in the number of leukocytes 10 days after surgery, as well as erythrocyturia throughout the entire period of monitoring. We consider the revealed differences are associated with the use of a modified three-way catheter, which contributed to a more thorough lavage of not only the bladder, but also the urethra, which is not available using a standard catheter, as well as the cytotoxic and immunosuppressive local action of 5-fluorouracil. A decrease in the process of cellular damage could play a positive role in the formation and growth of connective tissue, followed by the formation of strictures. The same effects can explain the statistically significant decrease in the level of prostate-specific antigen in the subjects of the main study group 10 days after surgery, when the inflammation process is most pronounced (Table 2).

An evaluation of the severity of dysuria symptoms in patients after surgery using the IPSS scale 10 days after the intervention and throughout the entire follow-up period showed a significant decrease in the total score both in the study group and in the control group. Patients noted a significant decrease in symptoms such as ischuria, stranguria, prolongation of the interval between urination, a decrease in episodes of intermittent urination, urinary incontinence, and straining before urination. Three months after the intervention, statistically significant differences were found in the main study group in comparison with the control. The positive effect of preventive intervention persisted six months after surgery (*p* < 0.001; 0.001, respectively) (Table 3).

Ten days after the operation, patients of both study groups noted an improvement in the quality of life. Differences in the study groups were borderline (χ^2^ = 9.31, *p* = 0.054). Three months after surgical treatment, differences in quality of life due to urination disorders in the study groups had statistically significant differences (χ^2^ = 37.53, *p* < 0.001). It was found that there was a significant increase in the number of people with an excellent and good quality of life in the main group, while in the group of patients who received standard procedures, persons with a satisfactory quality of life prevailed. The same trend persisted for six months after surgical treatment (χ^2^ = 40.935, *p* < 0.001) (Table 4).

Transurethral resection was performed with approximately the same frequency in both study groups. When comparing the average number of bed days of hospital stay in the main study group and the control group, no statistically significant differences were found (14 and 13 days, respectively) (*p* = 0.931).

According to the data presented in Table 5, the size of the prostate before surgery was statistically significantly larger in the control group (*p* = 0.04), while 6 months after surgery, its median size decreased from 58.0 to 16.0 cm^3^ in the main group and from 55.45 to 18.0 cm^3^ in the control group, while no statistically significant differences were observed in the study groups. Three and six months after the operation, there was a statistically significant decrease in the maximum urinary flow rate in the main group compared to the control group (*p* < 0.001; *p* < 0.001, respectively). In both study groups, after surgery, there was a positive dynamics of urine volume velocity throughout the study. The same trend was noted in relation to the amount of residual urine in patients of the study groups. Before the operation, this indicator was practically the same in both groups; after 10 days, it also did not have significant differences with an overall positive dynamics. After both 3 and 6 months, the rate in the main group was statically significantly lower than in the group with standard treatment, with overall positive dynamics in both groups (Table 5).

According to Table 6, the most pronounced positive dynamics were noted in relation to dysuria disorders in the form of pain and discomfort during urination. Ten days after the operation, both groups showed a significant improvement in the index; in the main study group, only a fifth of patients continued to experience symptoms of dysuria, while in the control group, the proportion of such patients was 36.1% (χ^2^ = 8.586; *p* = 0.003). A positive trend was noted throughout the entire follow-up period in both study groups, it was more pronounced in the main group (χ^2^ = 34.795; 39.261; *p* < 0.001; *p* < 0.001 after 3 and 6 months, respectively).

Table 7 presents a comparative description of urethral strictures in the studied patients. In the control group after three months, four cases of urethral strictures and stenosis were recorded; after six months, this rate reached nine cases (7.3%), while in the main group, only one patient with infravesical obstruction was found both three and six months after surgical treatment (0.8%) (χ^2^ = 3.855, *p* < 0.05).

After three months of observation, urethral bougienage was performed for four patients with established urethral strictures. One patient underwent optical urethrotomy; after six months, only one patient underwent urethral bougienage, and the rest of the patients underwent urethrotomy with urethral tunnelization.

To monitor the side effects of 5-fluorouracil, we analyzed a complete blood count and biochemical blood test on the first day after surgery, as well as 10 days and 1 month after surgery. There were no statistically significant changes in the levels of leukocytes, erythrocytes, platelets, and hemoglobin compared with the control group, although compared with the rates before surgery, both groups showed a decrease in the number of erythrocytes and hemoglobin and an increase in the platelet count, which is associated with intraoperative blood loss and postoperative microhematuria. With regard to renal function, there was a decrease in the median creatinine value in the main group from 100.0 before surgery to 82.2 mmol/L after 6 months and in the control group from 101.2 to 84.3 mmol/L, respectively.

## 4. Discussion

Prostate pathologies and androgen depletion are the most common conditions in men as they age. Several large cross-sectional studies have shown an annual decrease in testosterone levels in men with age of approximately 1–2% per year [15,16,17]. The presence of comorbidities plays a crucial role in reducing testosterone production. These include obesity, type 2 diabetes, and metabolic syndrome, which are accompanied by an increase in estrogen levels [18]. In men with BPH included in our study, type 2 diabetes mellitus occurred, on average in 15.4% (17.7% in the main study group and 13.1% in the control group) without a statistically significant difference, while as in the Republic of Kazakhstan, the prevalence of diabetes mellitus in 2017 reached 8.2%. Of interest is the fact that the metabolic syndrome is a predictor of the clinical progression of BPH [19], suggesting its role in the development of the disease.

There is very little information in the current literature on the clinical use of 5-fluorouracil in urological practice [20,21,22]. One of the mechanisms of action of 5-fluorouracil may involve the inhibition of type I collagen gene expression, which is induced by TGF-β (transforming growth factor beta, a protein representative of cytokines that controls proliferation and cell differentiation). Thus, the inhibition of excess collagen synthesis occurs similar to the action of corticosteroids [23]. Meanwhile, 5-fluorouracil is used to cover urethral stents used in cases of acute urinary retention in the presence of contraindications to surgery (strictures or stenosis of the prostatic or bulbar urethra, recurrent urethral strictures) [24].

In a study by Chinese scientists, using both in vivo and in vitro simulated urethral strictures, the effectiveness of the combined use of 5-fluorouracil and triamcinolone acetonide on the function of miRNAs in reducing the progression of strictures was studied. MiR-192-5p mediated drug-assisted urethral scar improvement by directly targeting ATG7, which is an autophagy marker gene. These studies have shown that triamcinolone in combination with 5-fluorouracil suppresses autophagy and fibroblasts in the urethral scar by increasing the expression of miP-192-5p [25].

In our study, taking into account the analysis of the literature data given above, we chose the use of 50 mg/mL 5-fluorouracil by irrigating the bladder and urethra using a modified three-way catheter as the main method of intervention to reduce the risk of developing a stricture or stenosis of the urethra. Analyzing the development of urethral strictures, we can note a positive trend three months after the operation, which means that in the main study group, we recorded only one patient with a formed urethral stricture throughout the entire follow-up period, while in the control group, such patients turned out to be four after 3 months and nine after 6 months. Statistically significant differences in the study groups were found after 6 months.

A comparative analysis of our data is difficult due to the lack of descriptions in the literature of the results of similar clinical studies on the prevention of urethral strictures in humans. However, they are fully consistent with the results of experimental studies and clinical studies evaluating the effectiveness of 5-fluorouracil in the treatment of keloid scars, as well as other cytostatics with antiproliferative activity (mitomycin C, corticosteroids) for the treatment and prevention of urethral strictures. With regard to the treatment of keloid scars, the efficacy of 5-fluorouracil was comparable to that of mitomycin C and bevacizumab and exceeded that of topical corticosteroids. Considering the significantly higher cost of treatment with mitomycin C, which is almost six times higher than that for 5-fluorouracil, with relatively the same effectiveness, we consider the choice of treatment tactics in our study justified. This is confirmed by the results of assessing the severity of dysuria symptoms using the IPSS rating scale [26].

Most patients with urethral stricture who undergo periodic self-dilatation consider their quality of life to be low. In a study of 85 patients with a mean age of 68 years, with intermittent self-dilatation once daily, the overall quality of life was rated as poor (mean score 7.0); young age, posterior urethral stricture, and catheterization difficulties were directly associated with a poor quality of life (*p* < 0.05; *p* = 0.04; *p* < 0.01, respectively) [27]. In a study conducted in Spain in 2018 on urethral plastic patients using the IPSS scale, it was found that the recurrence rate was 69%; the relative risk was 2.19. Quality of life was associated with the extent of the lesion (*p* = 0.046), symptoms of dysuria (*p* = 0.004), and individual self-assessment of health (*p* = 0.003). Lesion localization was associated with recurrence (*p* = 0.008) [28]. The effectiveness of using this scale has been confirmed in a number of other studies on the effectiveness of surgical interventions and the use of drugs for urethral strictures [29,30,31].

It has been established that urinary disorders in the long-term period after surgical treatment of BPH occur in 10–35% of patients [31,32]. The clinical manifestations of such disorders are increased urination, urge to urinate, difficulty urinating, and urinary incontinence [33]. With long-term urinary incontinence, aggravated by an increase in intra-abdominal pressure, it is necessary to think about possible damage to the external sphincter whose muscles are located concentrically behind the seminal tubercle. This symptom may be based on the anatomical features of hyperplastic tumor growth, leading to a diffuse dissection of the bladder sphincter. This feature can dramatically increase the risk of external sphincter fibers during the enucleation of hyperplastic adenoma tissues [34]. An evaluation of the effect of resectoscope diameter showed that the incidence of bulbar stricture was statistically significantly higher when using a 26 F instrument compared with a 24 F. However, maximum urine flow rate, IPSS, and postvoid residual urine volume were not dependent on resectoscope diameter. The authors concluded that the use of smaller diameter resectoscopes may lead to a reduction in the postoperative development of urethral strictures [35].

Most often, urination disorders are caused by detrusor hyperactivity, which disappears after the surgical removal of infravesical obstruction in 70–75% of patients [34]. In the absence of hyperactivity, the symptoms of dysuria persist in almost all patients even after repeated surgical intervention. In 16% of patients with dysuric disorders after adenomectomy, infravesical obstruction is observed, the cause of which is urethral stricture, recurrent prostate adenoma, and deformity of the bladder neck [33,34]. In our study, the use of 5-fluorouracil significantly reduced the symptoms of dysuria, as well as the amount of residual urine in comparison with patients receiving standard therapy.

## 5. Conclusions

The results of our study could indicate the effectiveness of the antiproliferative drug 5-fluorouracil in combination with the use of a modified catheter in relation to the development of postoperative urethral strictures. We can consider the effectiveness of this method in terms of a statistically significant reduction in the symptoms of dysuria, improvement in IPSS scores and quality of life of patients, improvement in urodynamics, as well as local inflammatory reactions in the urine. Considering that the study was conducted on a specific population, continued research in this field at the level of randomized multicenter clinical trials is necessary.

## Figures and Tables

**Table 1:** Characteristics of socio-demographic status and comorbidities of the studied persons.

Characteristics		N	%
Age (years)	40–50	1	0.4
	51–60	43	17.5
	61–70	119	48.4
	71–80	83	33.7
Body mass index	<25	77	31.3
	25–30	120	48.8
	>30	49	19.9
Residence	City	187	76.0
	Village	59	24.0
Type 2 diabetes		38	15.44
Arterial hypertension		93	37.8
Heart failure (I–II functional classes by NYHA)		21	8.53

**Table 2:** Comparative characteristics of laboratory parameters in the study groups.

Laboratory Rates	Study Groups						Test of Significance	
	Main Group			Control Group				
	Median	25th Percentile	75th Percentile	Median	25th Percentile	75th Percentile	U Test	*p*-Value
Urinalysis: leukocytes before surgery(in the field of view)	6.0	4.0	8.0	6.0	4.0	9.0	6902.5	0.233
10 days after surgery	6.0	5.0	8.5	8.0	5.0	10.0	6226.0	0.015
3 months after surgery	4.0	3.0	5.0	4.0	4.0	5.0	6730.5	0.125
6 months after surgery	3.0	2.0	4.0	3.0	2.0	4.0	7269.0	0.583
Urinalysis: erythrocytes before surgery (in the field of view)	5.0	3.0	8.0	5.0	3.0	7.0	7218.5	0.533
10 days after surgery	18.0	14.5	22.0	20.0	17.0	25.0	5715.5	<0.001
3 months after surgery	4.0	3.0	5.0	4.0	4.0	6.0	6357.5	0.026
6 months after surgery	2.0	1.0	2.5	2.0	2.0	3.0	5383.0	<0.001
Urinalysis: protein before surgery (g/L):	0.033	0.033	0.066	0.033	0.033	0.066	7329.5	0.648
10 days after surgery	0.33	0.165	0.99	0.33	0.165	0.99	7083.5	0.376
3 months after surgery	0.033	0.033	0.066	0.066	0.033	0.066	6883.0	0.174
6 months after surgery	0.033	0.033	0.033	0.033	0.033	0.033	6995.0	0.130
Prostate specific antigen before surgery (ng/L):	3.05	2.7	4.0	3.3	2.8	3.9	7394.0	0.760
10 days after surgery	4.0	3.3	4.4	4.1	3.3	4.6	6228.5	0.016
3 months after surgery	3.0	2.2	3.4	3.0	2.4	3.2	7408.0	0.779
6 months after surgery	2.6	2.1	3.0	2.45	2.1	2.9	7100.5	0.405

**Table 3:** Comparative characteristics of severity of urination disorders symptoms in accordance with IPSS scale.

Total Score	Study Groups						Test of Significance	
	Main Group			Control Group				
	Median	25th Percentile	75th Percentile	Median	25th Percentile	75th Percentile	U Test	*p*-Value
Before surgery	19	17	23	19	16	22	7057.5	0.362
10 days after surgery	12	10	16	14	11	15	7090.0	0.393
3 months after surgery	8	7	10	9	8	10	5703.0	<0.001
6 months after surgery	5	4	6	7	6	9	3573.5	<0.001

**Table 4:** Comparative characteristics of the patients’ quality of life in dynamics after surgery.

Quality of Life		Study Groups				Test of Significance	
		Main Group		Control Group			
		N	%	N	%	χ^2^	*p*-Value
Before surgery	Good	2	1.6	1	0.8	0.556	0.906
	Unsatisfactory	28	22.6	30	24.6		
	Bad	88	71.0	84	68.9		
	Very bad	6	4.8	7	5.7		
10 days after surgery	Beautiful	9	7.3	3	2.5	9.31	0.054
	Good	42	33.9	31	25.4		
	Satisfactory	66	53.2	73	59.8		
	Mixed feeling	7	5.6	12	9.8		
	Unsatisfactory	0	0.0	3	2.5		
3 months after surgery	Beautiful	8	6.5	2	1.6	37.530	<0.001
	Good	56	45.2	27	22.1		
	Satisfactory	58	46.8	64	52.5		
	Mixed feeling	2	1.6	29	23.8		
6 months after surgery	Beautiful	10	8.1	0	0.0	40.935	<0.001
	Good	64	51.6	29	23.8		
	Satisfactory	46	37.1	72	59.0		
	Mixed feeling	4	3.2	17	13.9		
	Unsatisfactory	0	0.0	4	3.3		

**Table 5:** Comparative characteristics of urodynamic parameters and prostate size in the study groups.

Rate	Study Groups						Test of Significance	
	Main Group			Control Group				
	Median	25th Percentile	75th Percentile	Median	25th Percentile	75th Percentile	U Test	*p*-Value
Prostate size before surgery (cm^3^)	58.0	51.95	64.7	55.45	48.4	62.3	6419.0	0.04
Prostate size 6 months after surgery	16.0	13.2	20.2	18.0	15.0	21.0	6551.0	0.069
Maximum urinary flow rate before surgery (mL/s)	7.0	5.0	9.0	7.0	6.0	9.0	7498.5	0.906
Maximum urinary flow rate 10 days after surgery (mL/s)	12.0	11.0	14.0	12.0	12.0	14.0	6984.0	0.290
Maximum urinary flow rate 3 months after surgery (mL/s)	20.0	18.0	22.0	18.0	15.0	20.0	4388.0	<0.001
Maximum urinary flow rate 6 months after surgery (mL/s)	22.0	20.0	23.0	19.0	18.0	21.0	3722.5	<0.001
The amount of residual urine before surgery (mL)	187.5	140.0	320.0	187.5	150.0	300.0	7558.5	0.992
The amount of residual urine 10 days after surgery (mL)	80.50	70.0	100.0	90.0	80.0	100.0	7134.5	0.437
The amount of residual urine 3 months after surgery (mL)	30.0	20.0	40.0	50.0	40.0	60.0	2860.0	<0.001
The amount of residual urine 6 months after surgery (mL)	20.0	10.0	30.0	40.0	30.0	50.0	3149.0	<0.001

**Table 6:** Comparative characteristics of dysuria in the studied groups before and after surgery.

Dysuria		Study Groups				Test of Significance	
		Main Group		Control Group			
		N	%	N	%	χ^2^	*p*-Value
Before surgery	Present	124	100.0	121	99.2	1.021	0.312
	Absent	-	-	1	0.8		
10 days after surgery	Present	24	19.4	44	36.1	8.586	0.003
	Absent	100	80.6	78	63.9		
3 months after surgery	Present	14	11.3	55	45.1	34.795	<0.001
	Absent	110	88.7	67	54.9		
6 months after surgery	Present	8	6.5	49	40.2	39.261	<0.001
	Absent	116	93.5	73	59.8		

**Table 7:** Comparative characteristics of development of urethral strictures in study groups after surgery.

Urethral Strictures		Study Groups				Test of Significance	
		Main Group		Control Group			
		N	%	N	%	χ^2^	*p*-Value
3 months after surgery	Present	1	0.8	4	3.3	1.888	0.169
	Absent	123	99.2	118	96.7		
6 months after surgery	Present	1	0.8	9	7.3	3.855	0.05
	Absent	123	99.2	113	92.7		
Stricture localization	Prostatic urethra	1	100	5	45		
	Membranous urethra	-	-	3	27		
	Bladder neck sclerosis	-	-	1	9		
Procedures 3 months after surgery	Bougienage	1	100	3	75		
	Urethrotomy	-	-	1	25		
Procedures 6 months after surgery	Bougienage	1	100	-	-		
	Urethrotomy	-	-	9	100		

## Data Availability

The datasets generated for this study are available on request to the corresponding author.
